# Rewiring Glycolysis in Cancer: From Tumor Initiation to Therapeutic Vulnerabilities

**DOI:** 10.3390/cells15090771

**Published:** 2026-04-24

**Authors:** Shicai Sun, Lulu Jia, Ying Yu, Seung-Jun Jeong, Yan Zhang, Dongryeol Ryu, Guang Ta

**Affiliations:** 1 Department of Surgery, Changchun University of Chinese Medicine, Changchun 130117, China; 2 Department of Biomedical Science and Engineering, Gwangju Institute of Science and Technology, Gwangju 61005, Republic of Korea

**Keywords:** glycolysis, cancer metabolism, metabolic rewiring, tumor progression, redox homeostasis, therapeutic vulnerability

## Abstract

**Highlights:**

**What are the main findings?**

- Glycolysis in cancer is not merely upregulated but dynamically rewired across different stages of tumorigenesis, with early metabolic changes actively contributing to malignant transformation rather than passively supporting tumor growth.
- Glycolytic rewiring integrates anabolic metabolism, redox homeostasis, and epigenetic regulation, and is continuously remodeled by transcriptional, post-translational, and microenvironmental inputs during tumor progression.

**What are the implications of the main findings?**

- Viewing glycolysis as a dynamic and stage-specific regulatory network provides a more comprehensive framework for understanding tumor metabolism and its role in coordinating multiple cancer-associated processes.
- The context-dependent metabolic dependencies generated by glycolytic rewiring highlight new opportunities for therapeutic targeting, particularly through strategies that exploit redox imbalance, metabolic plasticity, and combinatorial vulnerabilities.

**Abstract:**

Glycolysis is a defining feature of cancer metabolism, originally described by the Warburg effect. Increasing evidence indicates that cancer-associated glycolysis is not uniformly upregulated but dynamically rewired in response to oncogenic signaling, cellular demands, and microenvironmental cues. However, a framework integrating its temporal evolution and functional roles across tumorigenesis remains limited. In particular, how glycolytic rewiring drives malignant transformation, adapts during tumor progression, and generates context-dependent vulnerabilities has not been systematically synthesized. In this review, we examine glycolysis as a dynamic metabolic network evolving throughout tumor development. We discuss how early glycolytic rewiring, driven by oncogenic signaling and metabolic–epigenetic coupling, supports cell fate transitions and establishes redox and biosynthetic capacity during tumorigenesis. We then outline how glycolysis is remodeled during tumor progression through coordinated transcriptional, epigenetic, and post-translational regulation, as well as microenvironmental interactions and metabolic heterogeneity. Furthermore, we highlight glycolysis as an integrative hub linking immune evasion, cell death regulation, and metabolic plasticity, and discuss how glycolytic rewiring creates context-dependent metabolic dependencies that may be therapeutically exploited, along with emerging technologies that enable high-resolution characterization of tumor metabolism. Together, this review provides a conceptual framework for understanding glycolytic rewiring in cancer and outlines potential avenues for targeting metabolic vulnerabilities.

## 1. Introduction

Cancer metabolism has long been recognized as a defining feature of tumor biology. Nearly a century ago, the Warburg effect described the propensity of cancer cells to preferentially utilize glycolysis for energy production even in the presence of oxygen [1,2]. This phenomenon established aerobic glycolysis as a hallmark of cancer and laid the foundation for extensive investigations into metabolic alterations in tumor cells [3,4]. Subsequent studies have confirmed that elevated glycolytic activity supports rapid ATP generation and provides intermediates for anabolic processes, thereby facilitating sustained proliferation [5,6].

However, the classical view of the Warburg effect, which emphasizes a generalized increase in glycolytic flux, does not fully capture the complexity of metabolic regulation in cancer. Accumulating evidence indicates that glycolysis is not simply upregulated in a uniform manner but selectively and contextually reprogrammed. Distinct glycolytic enzymes, branch pathways, and metabolite pools are differentially controlled depending on oncogenic drivers, tumor stage, and microenvironmental conditions [7,8,9]. This suggests that cancer-associated glycolysis involves qualitative changes in pathway architecture and regulation, rather than a mere quantitative increase in activity.

In this context, the concept of metabolic rewiring has emerged as a more precise framework for understanding cancer metabolism. Glycolysis is dynamically rewired through coordinated regulation at multiple levels, including oncogenic signaling, transcriptional and epigenetic control, and post-translational modifications (PTMs) of metabolic enzymes [8,10,11]. These changes enable cancer cells to flexibly redirect metabolic flux toward biosynthesis, redox balance, and adaptive signaling, thereby supporting diverse cellular demands beyond energy production [12,13].

Importantly, glycolytic rewiring is a dynamic process that evolves throughout tumorigenesis. Early alterations in glycolytic regulation can facilitate cellular transformation by supporting anabolic growth and buffering oxidative stress [7,14]. As tumors progress, continued remodeling of glycolysis contributes to metabolic plasticity, interactions with the tumor microenvironment, and resistance to cell death [15,16]. These stage-specific adaptations not only sustain tumor development but also create context-dependent metabolic dependencies.

In this review, we discuss how glycolysis is dynamically rewired during tumor initiation and progression, and how these alterations give rise to specific therapeutic vulnerabilities. By integrating molecular mechanisms with functional consequences, we aim to provide a comprehensive framework for understanding glycolytic rewiring as both a driver of tumorigenesis and a targetable liability in cancer.

## 2. Tumor Initiation: Glycolytic Rewiring as a Driver of Cellular Transformation

### 2.1. Oncogenic Trigger of Glycolytic Rewiring

**Figure 1:**
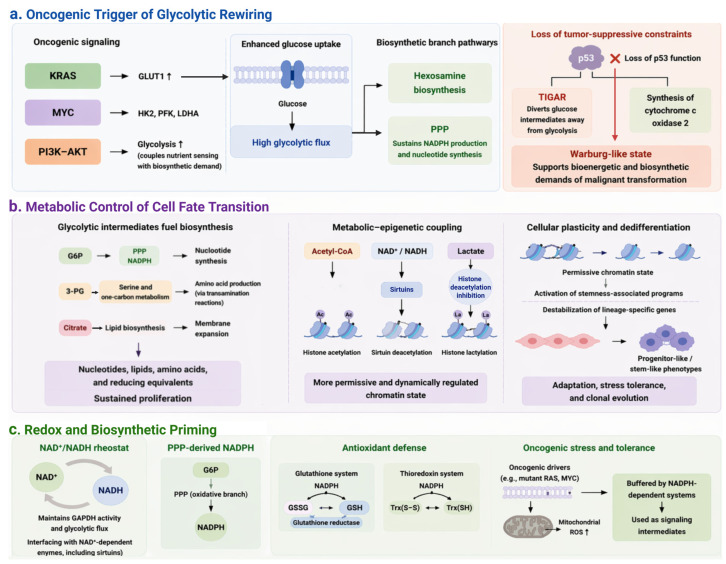
Early glycolytic rewiring drives tumor initiation and cell-state transition. (**a**) Oncogenic signaling driven by KRAS, MYC, and PI3K–AKT enhances glucose uptake and glycolytic gene expression, redirects intermediates into biosynthetic branch pathways, and, together with loss of p53 function, promotes a Warburg-like metabolic state that supports malignant transformation. Upward arrows indicate increased expression or flux. (**b**) Rewired glycolysis supplies intermediates for nucleotide, amino acid, and lipid synthesis and couples metabolism to chromatin regulation through acetyl-CoA, NAD^+^/NADH, and lactate, thereby enabling cellular plasticity and dedifferentiation. (**c**) Early glycolytic activation establishes redox and biosynthetic fitness by sustaining GAPDH activity and generating NADPH via the PPP, which supports glutathione- and thioredoxin-dependent antioxidant defense and enhances tolerance to oncogenic and metastatic stress. GLUT1, glucose transporter 1; HK2, hexokinase 2; PFK, phosphofructokinase; LDHA, lactate dehydrogenase A; TIGAR, TP53-induced glycolysis and apoptosis regulator; G6P, glucose-6-phosphate; 3-PG, 3-phosphoglycerate.

Glycolytic rewiring not only is an adaptation to the hypoxic tumor microenvironment or a consequence of uncontrolled proliferation, but also serves as an early, active driver of cellular transformation. This metabolic metamorphosis is orchestrated by a core circuitry of oncogenic signaling pathways, most notably Kirsten rat sarcoma (KRAS), myelocytomatosis (MYC), and the phosphoinositide 3-kinase (PI3K)–protein kinase B (AKT) axis, which collectively reprogram glucose flux at the very inception of tumorigenesis [17,18] (Figure 1a). At the earliest stage of transformation, convergent oncogenic signaling together with loss of tumor-suppressive constraints establishes a high-flux glycolytic state, characterized by enhanced glucose uptake and diversion of intermediates into biosynthetic branch pathways. This reprogramming establishes high glycolytic flux as a central metabolic node, enabling coordinated routing of glucose-derived carbon into anabolic and redox-supporting pathways.

Activation of KRAS oncogene serves as a master switch that enhances glucose uptake through the upregulation of glucose transporter 1 and concurrently shunts glycolytic intermediates into the hexosamine biosynthesis pathway and the pentose phosphate pathway (PPP) to satisfy anabolic requirements [19,20]. Meanwhile, transcription factor c-Myc acts as a global amplifier of the glycolytic transcriptome, directly transactivating nearly every gene in the glycolytic pipeline, including hexokinase 2 (HK2), phosphofructokinase (PFK), and lactate dehydrogenase A (LDHA), ensuring a high-velocity flux independent of oxygen availability [21,22,23]. Furthermore, the PI3K-AKT axis coordinates this metabolic surge by coupling extracellular nutrient sensing with intracellular biosynthetic demand, effectively locking cells into a state of constitutive glucose utilization [24,25]. This oncogenic gain-of-function is further reinforced by the loss of tumor-suppressive constraints. For instance, inactivation of p53, the most frequently mutated gene in human cancer, dismantles key metabolic checkpoints. Under physiological conditions, p53 restrains glycolytic flux in part through induction of TP53-induced glycolysis and apoptosis regulator (TIGAR), diverting glucose intermediates away from glycolysis, and supports mitochondrial respiration via the regulation of synthesis of cytochrome c oxidase 2 [26,27,28,29]. Conversely, loss of p53 function releases these metabolic constraints, biasing cells toward a Warburg-like state that is intrinsically configured to meet the bioenergetic and biosynthetic demands of malignant transformation. Collectively, this oncogenic circuitry defines the metabolic entry point of tumor initiation by coupling constitutive glycolytic activation with biosynthetic commitment.

### 2.2. Metabolic Control of Cell Fate Transition

Beyond its role in energy production, early glycolytic rewiring provides the essential metabolic framework for the profound cell fate transitions required for tumor initiation. Rather than functioning as a linear energy-producing pathway, glycolysis is strategically reprogrammed to redistribute carbon flux into multiple biosynthetic and regulatory branches (Figure 1b). This reprogramming can be functionally resolved into three interconnected modules: biosynthetic fueling, metabolic–epigenetic coupling, and the establishment of cellular plasticity.

Glycolytic intermediates support multiple biosynthetic pathways. Glucose-6-phosphate (G6P) is diverted into the PPP to sustain nucleotide synthesis and NADPH production. Meanwhile, 3-phosphoglycerate fuels serine and one-carbon metabolism. In addition, glycolysis contributes to lipid biosynthesis via citrate export and supports amino acid production through transamination reactions [30,31,32]. This coordinated allocation of metabolic intermediates simultaneously provides nucleotides for DNA replication, lipids for membrane expansion, and reducing equivalents to buffer oxidative stress, thereby enabling sustained proliferation during early neoplastic transformation [33,34]. Such coordinated metabolic routing establishes the biochemical foundation required for large-scale transcriptional and phenotypic reprogramming.

Importantly, the influence of glycolytic rewiring extends beyond biomass accumulation and directly interfaces with epigenetic regulation. Increasing evidence supports a tightly coupled and bidirectional relationship between metabolic flux and chromatin state. Glycolysis-derived metabolites regulate epigenetic enzymes. Acetyl-CoA serves as the donor for histone acetylation, linking nutrient availability to transcriptional activation. Changes in the NAD^+^/NADH ratio modulate sirtuin deacetylase activity, thereby coupling redox state to chromatin remodeling [35,36,37]. In parallel, lactate, traditionally viewed as a metabolic byproduct, has emerged as a signaling metabolite capable of inhibiting histone deacetylases and promoting histone lactylation, a recently characterized modification associated with gene activation [38]. Through these mechanisms, elevated glycolytic flux establishes a permissive and dynamically regulated chromatin environment. This, in turn, enables rapid transcriptional reprogramming in response to oncogenic signals. This metabolic–epigenetic coupling plays a central role in the acquisition of cellular plasticity. By reshaping chromatin accessibility and transcriptional networks, glycolysis facilitates the activation of stemness-associated programs while destabilizing lineage-specific gene expression [39,40]. Meanwhile, the integration of glycolytic metabolism with one-carbon pathways influences methyl donor availability, thereby impacting DNA and histone methylation patterns that further reinforce cell state transitions. As a result, committed somatic cells gain the capacity to undergo dedifferentiation and adopt progenitor-like or stem-like phenotypes [41,42].

Such phenotypic reversion is a hallmark of early tumorigenesis and is essential for adaptation to the evolving tumor microenvironment. Glycolysis-driven plasticity enables cells to tolerate fluctuations in oxygen and nutrient availability, resist differentiation cues, and evade apoptosis induced by oncogenic or metabolic stress [43]. Moreover, this flexible state allows for rapid selection of advantageous phenotypes under selective pressure, thereby accelerating clonal evolution at early stages of tumor development. Glycolysis functions as an integrative regulatory hub coordinating anabolic metabolism, redox balance, and epigenetic remodeling to control cell identity. Through this multifaceted role, glycolytic rewiring transforms a stable, differentiated cell into a metabolically adaptable and transcriptionally plastic entity, thereby laying the foundational groundwork for malignant transformation. Collectively, these processes illustrate how glycolytic rewiring drives the transition from metabolic activation to cell fate plasticity.

### 2.3. Redox and Biosynthetic Priming

A critical yet underappreciated dimension of early glycolytic rewiring lies in its capacity to precondition the intracellular milieu for sustained oxidative stress and the escalating anabolic demands of transformation. Rather than serving merely as an adaptive response to hypoxia or rapid proliferation, the early activation of glycolysis constitutes a proactive metabolic reprogramming event that establishes redox resilience and biosynthetic competence [4,44] (Figure 1c). This stage extends glycolytic function beyond anabolic support by establishing a redox-buffered intracellular environment that enables sustained tolerance to oncogenic stress.

Central to this process is the tight regulation of the NAD^+^/NADH ratio, which functions as a key metabolic rheostat governing both glycolytic flux and the broader cellular redox landscape [45]. This rheostat links glycolytic activity to both redox balance and the activity of NAD^+^-dependent regulatory enzymes, thereby coordinating metabolic and signaling processes. The maintenance of NAD^+^ availability ensures continuous operation of glyceraldehyde-3-phosphate dehydrogenase, thereby sustaining high glycolytic throughput, while also interfacing with multiple NAD^+^-dependent enzymes, including sirtuins, that coordinate stress responses and chromatin dynamics [46,47]. Meanwhile, the rerouting of G6P into the oxidative branch of the PPP represents a pivotal node in this metabolic reconfiguration. Through the activity of G6P dehydrogenase, transforming cells generate substantial quantities of NADPH, a critical reducing equivalent that underpins antioxidant defense systems [48,49]. NADPH is indispensable for maintaining glutathione in its reduced form via glutathione reductase, as well as for sustaining thioredoxin-dependent redox cycling [50]. These systems collectively buffer intracellular reactive oxygen species (ROS), thereby preventing oxidative damage to DNA, lipids, and proteins while preserving cellular integrity under oncogenic stress. This redox buffering capacity represents a critical enabling condition that allows transformed cells to tolerate otherwise cytotoxic levels of oxidative and metabolic stress. During the earliest phases of cellular transformation, activation of oncogenic drivers, such as mutant RAS or MYC, frequently induces a burst of mitochondrial ROS as a byproduct of altered electron transport chain dynamics [44,51]. In the absence of a compensatory increase in antioxidant capacity, such ROS accumulation would trigger DNA damage responses, senescence, or apoptosis. Glycolytic rewiring, however, effectively offsets this threat by establishing a robust NADPH-dependent redox buffering system. This enables cells not only to tolerate elevated ROS levels but also to exploit them as signaling intermediates [48].

Beyond immediate redox homeostasis, this early metabolic adaptation exerts long-lasting effects on tumor evolution by establishing a form of metabolic memory or dependency. Cells that have undergone glycolytic priming become intrinsically reliant on sustained glucose flux and NADPH production to maintain redox balance and biosynthetic output [52]. This dependency confers a selective advantage under subsequent stress conditions encountered during tumor progression. For instance, during detachment from the extracellular matrix, cells experience elevated oxidative stress that would normally induce anoikis; however, preconditioned cells can resist this fate through enhanced antioxidant capacity. Similarly, during metastasis and colonization of distant tissues-where nutrient and oxygen availability are often limited-this pre-established metabolic flexibility supports survival and outgrowth [53,54]. Collectively, early glycolytic rewiring should be viewed not simply as a metabolic hallmark of cancer, but as a foundational event that integrates redox control, biosynthetic readiness, and stress adaptability. By simultaneously securing ATP production, generating anabolic precursors, and fortifying antioxidant defenses, this reprogramming enables cells to navigate the multiple selective pressures of tumorigenesis. In doing so, it establishes a robust and evolvable metabolic framework that underlies malignant progression and therapeutic resistance. Collectively, glycolysis-derived reducing equivalents not only neutralize oxidative damage but also actively shape redox-dependent signaling during transformation.

Taken together, early glycolytic rewiring integrates oncogenic activation, metabolic–epigenetic coupling, and redox priming into a unified and staged framework that underpins tumor initiation (Figure 1).

## 3. Tumor Progression: Dynamic Remodeling of Glycolytic Networks

**Figure 2:**
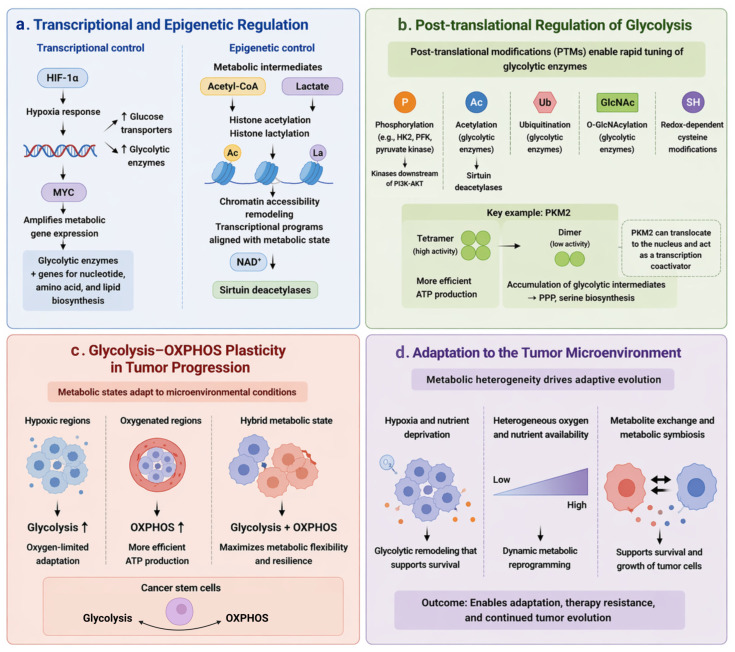
Multilayer regulation and metabolic plasticity of glycolysis during tumor progression. (**a**) Glycolytic remodeling during tumor progression is reinforced by transcriptional and epigenetic programs. HIF-1α and MYC enhance glucose uptake and glycolytic gene expression, while also coordinating biosynthetic gene programs. Metabolic intermediates reshape chromatin states. (**b**) PTMs, including phosphorylation, acetylation, ubiquitination, O-GlcNAcylation, and redox-dependent cysteine modifications, dynamically regulate glycolytic enzyme activity. PKM2 functions as a central node by interconverting between tetrameric and dimeric states and by exerting non-metabolic nuclear functions. (**c**) Cancer cells adapt to microenvironmental changes by switching between glycolysis and OXPHOS by maintaining hybrid metabolic states. (**d**) Metabolic flexibility, together with intratumoral heterogeneity and metabolite exchange, supports survival, stem-like properties, and continued tumor evolution under stress conditions. HIF-1α, hypoxia-inducible factor 1 alpha; PTMs, post-translational modifications; PKM2, pyruvate kinase M2. Upward arrows indicate increased expression or flux.

As tumors progress, glycolytic metabolism undergoes continuous and dynamic remodeling to accommodate increasing demands for growth, survival, and adaptation. Rather than remaining fixed after initial transformation, glycolysis is persistently reshaped by both intrinsic regulatory programs and extrinsic environmental pressures. This evolving metabolic landscape enables tumor cells to sustain proliferation, tolerate stress, and adapt to heterogeneous microenvironments. This dynamic remodeling can be conceptualized as a multi-layered regulatory framework encompassing transcriptional and epigenetic control, post-translational modulation, metabolic state transitions, and microenvironment-driven adaptation (Figure 2).

### 3.1. Transcriptional and Epigenetic Regulation

During tumor progression, transcriptional and epigenetic mechanisms play central roles in sustaining and refining glycolytic rewiring (Figure 2a). Among these, hypoxia-inducible factor 1α (HIF-1α) serves as a master regulator of the hypoxic response, orchestrating a coordinated transcriptional program that enhances glucose uptake and glycolytic flux [55,56]. By directly upregulating genes encoding glucose transporters and key glycolytic enzymes, HIF-1α ensures metabolic continuity under oxygen-limited conditions while simultaneously suppressing mitochondrial oxidative metabolism [57,58]. This shift not only preserves ATP production but also minimizes excessive mitochondrial ROS generation, thereby contributing to redox balance. In parallel, oncogenic transcription factors such as MYC act as global amplifiers of metabolic gene expression, reinforcing and expanding the glycolytic program initiated by hypoxia signaling [59,60]. MYC-driven transcription extends beyond canonical glycolytic enzymes to include genes involved in nucleotide, amino acid, and lipid biosynthesis, thereby integrating glycolysis into a broader anabolic network. This transcriptional control is complemented by metabolic intermediates, including acetyl-CoA, lactate, and NAD^+^, which directly link glycolytic activity to chromatin remodeling. The cooperative interaction between HIF-1α and MYC thus establishes a robust and multilayered transcriptional framework that supports both metabolic flux and biosynthetic capacity, even under adverse environmental conditions [61,62].

Beyond transcriptional control, epigenetic regulation provides an additional and increasingly recognized layer of metabolic governance. A defining feature of this regulation is the direct coupling of metabolic intermediates to chromatin-modifying processes. Glycolysis-derived metabolites, such as acetyl-CoA and lactate, function as substrates or signaling molecules for histone modifications, including histone acetylation and histone lactylation [37,63]. These modifications dynamically reshape chromatin accessibility and transcriptional outputs, enabling cancer cells to align gene expression programs with metabolic state. Furthermore, the availability of cofactors such as NAD^+^ links cellular redox status to the activity of epigenetic regulators, including sirtuin family deacetylases. Through these mechanisms, metabolic flux is translated into epigenetic information, effectively creating a feedback loop in which glycolysis both regulates and is reinforced by chromatin state [64,65,66]. This bidirectional crosstalk stabilizes glycolytic phenotypes while preserving sufficient plasticity to accommodate environmental changes. Through these mechanisms, glycolysis is both regulated by and reinforces chromatin accessibility and transcriptional programs aligned with metabolic state.

Collectively, the integration of transcriptional and epigenetic control mechanisms allows tumor cells to fine-tune glycolytic activity with remarkable precision. This multilayered regulatory architecture not only sustains metabolic reprogramming but also endows cancer cells with the adaptability required for continued evolution, therapeutic resistance, and progression toward more malignant states.

### 3.2. Post-Translational Regulation of Glycolysis

In contrast to transcriptional and epigenetic mechanisms that operate on relatively slower timescales, PTMs enable tumor cells to dynamically recalibrate enzymatic activity in response to acute fluctuations in nutrient availability, redox status, and signaling inputs [67,68] (Figure 2b). Glycolytic enzymes are tightly regulated by covalent modifications, including phosphorylation, acetylation, ubiquitination, O-GlcNAcylation, and redox-dependent cysteine modifications. These modifications control catalytic efficiency, protein stability, subcellular localization, and interactions with binding partners [69,70].

Among these regulatory paradigms, phosphorylation-mediated control of glycolytic enzymes represents a key interface between oncogenic signaling pathways and metabolic flux. Kinases downstream of pathways such as PI3K-AKT and MAPK directly modify enzymes including HK2, PFK, and pyruvate kinase, thereby rapidly adjusting glycolytic throughput to support proliferative demands [71,72,73]. A particularly well-characterized example is the functional modulation of pyruvate kinase M2 (PKM2), which exists in distinct oligomeric states with differential catalytic activity [74]. The tetrameric form is highly active and efficiently converts phosphoenolpyruvate into pyruvate, generating ATP. In contrast, the less active dimeric form leads to the accumulation of upstream glycolytic intermediates. These intermediates can then be redirected into anabolic pathways, including the PPP and serine biosynthesis [75,76]. This regulated activity switch allows cancer cells to balance bioenergetic needs with the generation of macromolecular precursors, thereby optimizing metabolic output for growth. Acetylation-dependent regulation links enzyme function to cellular nutrient and energy status by modulating catalytic activity, stability, and susceptibility to degradation, while NAD^+^-dependent deacetylases such as SIRT1 and SIRT2 act as metabolic sensors that couple redox state to enzyme regulation [77,78]. Through this mechanism, fluctuations in the NAD^+^/NADH ratio are translated into coordinated changes in glycolytic flux, effectively integrating cellular energy balance with metabolic pathway activity. Beyond acetylation, other metabolite-sensitive PTMs-such as O-GlcNAcylation, which reflects glucose availability through the hexosamine biosynthetic pathway-further contribute to the fine-tuning of glycolysis in response to nutrient cues [79,80]. In addition to modulating enzymatic activity, PTMs also govern the spatial organization of glycolytic enzymes, thereby adding a critical layer of compartmentalized regulation. Increasing evidence indicates that several glycolytic enzymes can translocate to the nucleus or associate with specific subcellular structures, where they perform noncanonical, “moonlighting” functions. For instance, PKM2 can translocate to the nucleus and act as a coactivator of transcription factors, influencing the expression of genes involved in proliferation and metabolic adaptation [81]. Collectively, post-translational regulation endows glycolysis with a high degree of plasticity and responsiveness, enabling tumor cells to rapidly adapt metabolic outputs to evolving environmental and intracellular conditions. By integrating signaling pathways, redox state, and nutrient availability at the level of enzyme function and localization, these mechanisms ensure that glycolytic flux remains optimally tuned to support tumor progression.

Notably, PTMs do not operate with equal functional weight under all conditions. Rather, a hierarchical organization exists in which certain PTMs function as basal regulators that fine-tune enzymatic activity, whereas others act as dominant, context-dependent “master switches” in response to specific environmental pressures.

Under hypoxic conditions, phosphorylation-dependent signaling pathways are preferentially engaged to rapidly enhance glycolytic flux and support adaptation to oxygen limitation. In contrast, nutrient availability and cellular energy status are more closely linked to acetylation and NAD^+^-dependent deacetylation, which coordinate metabolic activity with redox balance and mitochondrial function [82,83]. Similarly, O-GlcNAcylation serves as a nutrient-sensitive modification that integrates glucose flux through the hexosamine biosynthetic pathway with enzyme activity and signaling networks [84,85]. This hierarchical deployment of PTMs allows cancer cells to dynamically prioritize specific regulatory mechanisms depending on environmental context, thereby enabling both rapid metabolic adjustment and sustained adaptation.

### 3.3. Metabolic Plasticity and Intratumoral Heterogeneity

As tumors evolve, metabolic plasticity emerges as a defining, cell-intrinsic property that enables cancer cells to dynamically reprogram their metabolic states (Figure 2c). Rather than relying exclusively on glycolysis, tumor cells retain the capacity to transition between glycolysis, oxidative phosphorylation (OXPHOS), and intermediate metabolic configurations. This flexibility is governed by oncogenic signaling and regulatory networks, allowing cancer cells to maintain metabolic adaptability. These states include glycolysis-dominant metabolism in hypoxic regions, OXPHOS-dominant metabolism in oxygenated regions, and hybrid metabolic states that integrate both pathways.

In addition, metabolic plasticity underlies the emergence of intratumoral metabolic heterogeneity. Distinct tumor subpopulations can adopt different metabolic preferences, reflecting variations in signaling states, epigenetic regulation, and cellular hierarchy. Such diversity is not merely a byproduct of tumor evolution but represents a functional extension of metabolic plasticity at the population level. Cancer stem cells (CSCs) exemplify this adaptability. These cells can reversibly engage glycolytic and oxidative programs to support self-renewal and long-term survival [86,87]. Their metabolic flexibility contributes to persistence under stress and therapeutic challenge, highlighting the role of plasticity-driven heterogeneity in shaping tumor behavior.

### 3.4. Microenvironment-Driven Metabolic Adaptation

While metabolic plasticity defines the intrinsic capacity, microenvironmental adaptation reflects the context-dependent deployment of this flexibility. In this framework, metabolic plasticity defines what tumor cells can do, whereas microenvironmental adaptation determines when and how specific metabolic states are deployed. Rather than being constrained to a fixed metabolic program such as aerobic glycolysis, cancer cells occupy a dynamic metabolic spectrum, characterized by the context-dependent utilization of glycolysis, OXPHOS, or hybrid metabolic states [88,89] (Figure 2d). This adaptability is not stochastic but is tightly regulated by both cell-intrinsic signaling networks and extrinsic environmental cues. Hypoxia and nutrient deprivation, heterogeneous oxygen and nutrient availability, and metabolite exchange between tumor subpopulations collectively drive this adaptive metabolic remodeling. Fluctuations in oxygen tension, nutrient availability, and stromal interactions continuously reshape metabolic demands, necessitating rapid and reversible adjustments in energy production and biosynthetic flux. Crucially, context-dependent engagement of glycolysis or OXPHOS provides a selective advantage under conditions of metabolic stress. In hypoxic or nutrient-deprived regions, enhanced glycolysis supports ATP generation and redox balance, whereas in more oxygenated niches, reliance on mitochondrial oxidative metabolism enables more efficient ATP production and supports processes such as fatty acid oxidation and ROS-mediated signaling. Increasing evidence suggests that many cancer cells adopt a hybrid metabolic phenotype, simultaneously engaging glycolysis and OXPHOS to maximize metabolic flexibility and resilience [90,91]. This hybrid state allows tumor cells to maintain energetic homeostasis while preserving the capacity to rapidly redirect metabolic intermediates toward anabolic or stress-adaptive pathways.

In parallel, intratumoral heterogeneity further amplifies metabolic diversity, giving rise to distinct subpopulations with specialized metabolic phenotypes. Genetic variation, epigenetic divergence, and microenvironmental gradients collectively drive the emergence of metabolically distinct tumor clones. For example, cells located in hypoxic tumor cores often exhibit a highly glycolytic phenotype, whereas those at the invasive front or in vascularized regions may preferentially utilize OXPHOS [92]. This spatial metabolic compartmentalization not only supports differential proliferative and survival capacities but also facilitates cooperative interactions between tumor subpopulations, including metabolite exchange and metabolic symbiosis. Such interactions enable metabolite exchange between glycolytic and oxidative tumor cell populations, supporting overall tumor survival and growth. Notably, shifts toward OXPHOS have been associated with enhanced resistance to chemotherapy and radiotherapy, partly due to improved mitochondrial function and redox control, whereas glycolytic states may support rapid proliferation and niche colonization [93,94]. The ability of CSCs to reversibly reprogram their metabolism thus represents a critical mechanism driving tumor recurrence and disease progression. Metabolic plasticity and heterogeneity are not merely byproducts of tumor evolution but active drivers of malignant progression. By enabling cancer cells to navigate fluctuating environmental constraints and therapeutic challenges, these properties facilitate the selection of resilient and adaptable cellular populations. Understanding the regulatory mechanisms that govern metabolic state transitions and heterogeneity will therefore be essential for the development of strategies aimed at targeting metabolic vulnerabilities and overcoming therapy resistance in cancer.

Taken together, these findings indicate that glycolytic remodeling during tumor progression is coordinated across transcriptional, post-translational, metabolic, and microenvironmental levels, collectively supporting tumor adaptation and evolution (Figure 2).

## 4. Functional Integration: Glycolysis as a Central Hub of Cancer Hallmarks

Beyond its canonical roles in ATP production and the provision of biosynthetic intermediates, glycolysis functions as a central integrative hub that coordinates multiple hallmarks of cancer. Rather than operating as an isolated metabolic pathway, glycolysis generates a network of intermediate and redox cofactors that interface with signaling pathways, epigenetic regulators, and microenvironmental dynamics. These processes are not independent; instead, they are interconnected through shared metabolic dependencies and feedback loops. As a result, glycolysis acts as a functional nexus that synchronizes cellular behavior with environmental constraints, thereby reinforcing tumor progression, adaptability, and therapeutic resistance.

### 4.1. Immune Evasion

**Figure 3:**
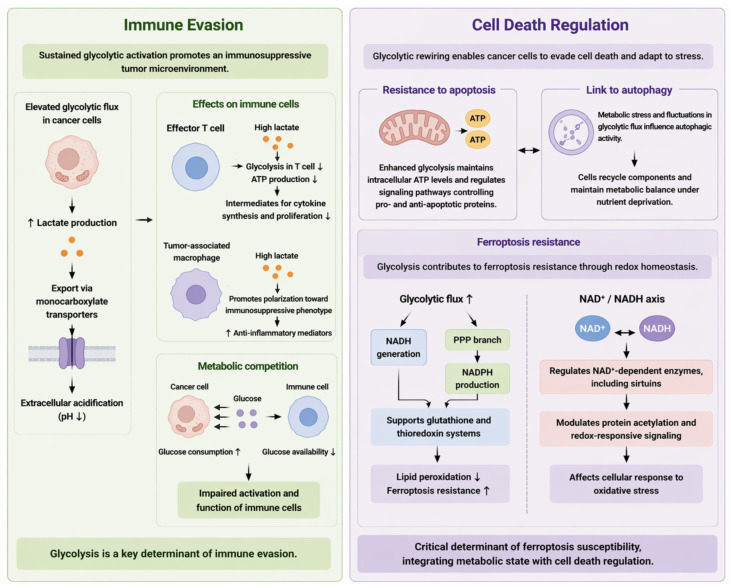
Glycolytic rewiring links immune evasion to cell-death regulation. Sustained glycolytic activation remodels the tumor microenvironment and promotes immune evasion by increasing lactate production and export via monocarboxylate transporters, leading to extracellular acidification, suppression of effector T-cell metabolism, and polarization of tumor-associated macrophages toward an immunosuppressive phenotype. In parallel, glycolytic rewiring supports resistance to apoptosis, interacts with autophagy under nutrient stress, and contributes to ferroptosis resistance by maintaining redox homeostasis through the NAD^+^/NADH axis and NADPH production via the PPP to sustain glutathione and thioredoxin systems. Together, these processes position glycolysis as an integrative metabolic hub linking immune suppression with redox-dependent cell-death regulation. ATP, adenosine triphosphate; NADPH, reduced nicotinamide adenine dinucleotide phosphate. Upward arrows indicate increased expression or flux; downward arrows indicate decreased activity or availability.

One of the most prominent consequences of sustained glycolytic activation is the establishment of an immunosuppressive tumor microenvironment (Figure 3). Elevated glycolytic flux drives the excessive production of lactate, which is actively exported via monocarboxylate transporters, leading to extracellular acidification [44,95]. This lactate accumulation and extracellular acidification represent key metabolic outputs of elevated glycolytic flux that directly reshape the tumor microenvironment. This metabolic reprogramming exerts profound effects on immune cell function at multiple levels, effectively reshaping anti-tumor immunity. High extracellular lactate concentrations impair the function of effector T cells by disrupting their metabolic fitness. Specifically, lactate accumulation inhibits glycolysis within T cells, thereby limiting ATP production and reducing the availability of intermediates required for cytokine synthesis and proliferation [96,97]. Meanwhile, lactate acts as a signaling metabolite that promotes the polarization of tumor-associated macrophages toward an immunosuppressive, pro-tumorigenic phenotype, characterized by enhanced expression of anti-inflammatory mediators [98,99]. This polarization is associated with increased secretion of anti-inflammatory factors that further suppress anti-tumor immunity.

Beyond lactate-mediated effects, metabolic competition within the tumor microenvironment represents an additional mechanism of immune suppression. Highly glycolytic cancer cells consume large amounts of glucose, thereby depriving infiltrating immune cells of essential nutrients required for their activation and function [100,101]. This competition reduces glucose availability for immune cells, further limiting their activation and metabolic fitness. This competition creates a metabolically hostile niche in which immune cells are unable to sustain effective anti-tumor responses. Collectively, these mechanisms position glycolysis as a key determinant of immune evasion, linking metabolic reprogramming directly to the suppression of immune surveillance.

### 4.2. Cell Death Regulation

Glycolytic rewiring also plays a central role in modulating cell death pathways, enabling cancer cells to evade lethal stress while preserving metabolic adaptability (Figure 3). In this context, glycolysis integrates bioenergetic support with redox regulation to influence multiple cell death pathways. Enhanced glycolysis supports resistance to apoptosis through multiple mechanisms, including the maintenance of intracellular ATP levels and the regulation of signaling pathways that control pro- and anti-apoptotic proteins [102]. Sustained ATP production through glycolysis supports survival signaling and suppresses apoptotic activation. In parallel, glycolysis is intricately linked to autophagy, a catabolic process that supports cell survival under nutrient deprivation. Metabolic stress and fluctuations in glycolytic flux influence autophagic activity, allowing cancer cells to recycle intracellular components and maintain metabolic balance [103,104]. This coupling enables cells to recycle intracellular components and sustain metabolic homeostasis under nutrient stress. This coordination between glycolysis and autophagy provides a survival advantage in fluctuating microenvironments.

Notably, emerging evidence has highlighted a critical intersection between glycolysis and ferroptosis, a form of regulated cell death driven by iron-dependent lipid peroxidation. Glycolysis contributes to ferroptosis resistance primarily through its control of cellular redox homeostasis [105]. Increased glycolytic flux supports the generation of reducing equivalents, both directly via NADH and indirectly through diversion of glucose into the PPP, which produces NADPH [106]. These reducing equivalents sustain glutathione and thioredoxin systems, thereby suppressing lipid peroxidation and increasing ferroptosis resistance. Meanwhile, the NAD^+^/NADH axis serves as a key regulatory node linking glycolysis to ferroptosis sensitivity. NAD^+^ availability influences the activity of NAD^+^-dependent enzymes, including sirtuins, which modulate protein acetylation status and redox-responsive signaling pathways [107]. Through modulation of NAD^+^-dependent enzymes, glycolysis links metabolic state to protein acetylation and redox-responsive signaling. Through these mechanisms, alterations in glycolytic activity can reshape NAD^+^ metabolism, thereby affecting downstream deacetylation processes and the cellular response to oxidative stress. Consequently, glycolysis functions as a critical determinant of ferroptosis susceptibility, integrating metabolic state with cell death regulation.

Taken together, glycolysis coordinates immune suppression and regulation of apoptosis, autophagy, and ferroptosis, thereby integrating metabolic state with tumor survival and stress adaptation (Figure 3).

## 5. The Dichotomous Role of Glycolysis: Metabolic Constraints and Context-Dependent Anti-Tumor Effects

While the canonical view emphasizes the tumor-promoting aspects of the Warburg effect, emerging evidence suggests that the glycolytic phenotype is not universally advantageous and can, under specific contexts, impose significant biological costs or even exert inhibitory effects on tumorigenesis. Sustained glycolytic activation may introduce intrinsic constraints that limit metabolic flexibility, amplify cellular stress, and expose context-dependent vulnerabilities.

### 5.1. Metabolic Vulnerabilities

The energetic inefficiency of glycolysis creates an absolute requirement for high-rate glucose uptake, a phenomenon termed “glucose addiction” [108,109]. In the nutrient-deprived cores of solid tumors where angiogenesis is impaired, this dependency becomes a survival bottleneck. Recent studies have demonstrated that excessive glycolytic flux can deplete intracellular pools of non-glycolytic intermediates, leading to a “metabolic trap” where cells lack the flexibility to switch to OXPHOS under acute stress [110,111]. Notably, this dependence extends beyond carbon flux to redox homeostasis. Sustained glycolytic activity requires continuous regeneration of NAD^+^ to maintain glyceraldehyde-3-phosphate dehydrogenase activity, thereby coupling glycolytic throughput to NAD^+^ availability. In highly glycolytic cells, disruption of NAD^+^ recycling, such as inhibition of LDH-mediated NAD^+^ regeneration, has been shown to rapidly impair glycolytic flux and induce energetic and redox collapse, ultimately triggering cell death [112]. These observations reveal a fundamental trade-off inherent to sustained glycolytic rewiring, whereby the prioritization of high glycolytic throughput imposes a strict dependence on both substrate availability and redox recycling. This dual dependency constrains metabolic flexibility and the capacity of tumor cells to re-engage mitochondrial respiration under stress conditions. As such, highly glycolytic cells become selectively vulnerable to metabolic perturbations, including glucose deprivation or redox imbalance, which may precipitate cell death programs such as ferroptosis [113].

### 5.2. Pro-Inflammatory Signaling and Immune Recognition

Contrary to the immune-suppressive role of lactate, intermediates of the glycolytic pathway can, under certain conditions, serve as metabolic signals that promote anti-tumor immunity. For instance, specific glycolytic intermediates or their non-metabolic functions have been reported to trigger dendritic cell maturation [114,115]. In addition, enhanced glycolytic activity in certain tumor contexts has been associated with increased expression of MHC-I molecules and interferon-responsive genes, potentially improving tumor immunogenicity [116,117]. Collectively, these findings highlight a paradoxical pro-immunogenic facet of glycolysis and further support the notion that its immunological impact is context-dependent rather than uniformly immunosuppressive.

### 5.3. Feedback Inhibition and Intracellular Toxicity

The aggressive pursuit of glycolysis leads to the accumulation of reactive byproducts beyond lactate, such as methylglyoxal, a potent dicarbonyl stressor. While it can promote mutations, its excessive accumulation is toxic, inducing proteotoxicity and DNA damage that can lead to metabolic catastrophe [118,119]. Furthermore, the localized intracellular acidification caused by rapid proton generation can inhibit rate-limiting glycolytic enzymes like PFK1, creating a self-limiting feedback loop. This proton burden imposes an intrinsic biochemical constraint on glycolytic throughput, preventing indefinite flux escalation. In certain genetic backgrounds, this acidification triggers the intrinsic apoptotic pathway rather than facilitating invasion, effectively acting as a metabolic brake on tumor progression [120].

## 6. Therapeutic Vulnerabilities: Targeting Rewired Glycolysis

**Figure 4:**
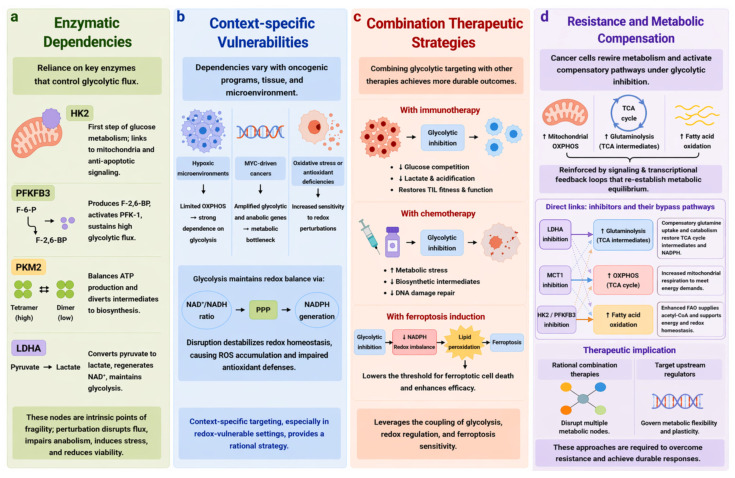
Therapeutic vulnerabilities created by rewired glycolysis in cancer. (**a**) Rewired glycolysis creates selective dependence on key enzymatic nodes that control metabolic flux, including HK2, PFKFB3, PKM2, and LDHA; perturbation of these enzymes disrupts flux distribution, impairs anabolic capacity, and compromises tumor cell viability. (**b**) Glycolytic targeting is context-dependent, as reliance on this pathway varies with oncogenic programs, hypoxia, and redox status; tumors with limited OXPHOS capacity, MYC activation, or antioxidant vulnerability are particularly sensitive, as glycolysis supports redox balance through the NAD^+^/NADH axis and NADPH production via the PPP. (**c**) Combining glycolytic inhibition with immunotherapy, chemotherapy, or ferroptosis induction enhances therapeutic efficacy by relieving metabolic competition, increasing metabolic stress, and lowering the threshold for lipid peroxidation-driven cell death. (**d**) Tumor cells can evade glycolytic inhibition through metabolic compensation, including increased OXPHOS, glutaminolysis, and fatty acid oxidation. Notably, specific glycolytic perturbations may preferentially engage distinct bypass pathways; for example, LDHA inhibition can promote compensatory glutaminolysis to sustain TCA cycle intermediates and redox balance, whereas MCT1 inhibition may enhance reliance on mitochondrial OXPHOS, and inhibition of upstream glycolytic nodes such as HK2 or PFKFB3 can increase fatty acid oxidation. These adaptive responses highlight the need for rational combination strategies or targeting of upstream regulators to overcome metabolic resistance. PFKFB3, 6-phosphofructo-2-kinase/fructose-2,6-bisphosphatase 3; TCA cycle, tricarboxylic acid cycle. Upward arrows indicate increased expression or flux; downward arrows indicate decreased ac-tivity or availability.

The dynamic rewiring of glycolysis during tumorigenesis not only fuels malignant progression but also engenders distinct metabolic dependencies that can be therapeutically exploited. Rather than representing a uniform metabolic liability, these vulnerabilities emerge from the convergence of enzyme-level control points, context-specific metabolic states, and constraints on adaptive capacity under targeted perturbation. Importantly, however, the therapeutic window for targeting glycolysis is intrinsically shaped by the heterogeneity and plasticity of tumor metabolism. Effective intervention therefore requires a nuanced understanding of how glycolytic dependencies are established, maintained, and remodeled during disease progression. These therapeutic vulnerabilities can be broadly categorized into enzymatic dependencies, context-specific metabolic states, combination strategies, and resistance-associated metabolic compensation (Figure 4).

### 6.1. Enzymatic Dependencies

Rewired glycolysis imposes a selective reliance on a limited set of enzymatic nodes that exert disproportionate control over metabolic flux. Among these, HK2, PFK2/fructose-2,6-bisphosphatase 3 (PFKFB3), PKM2, and LDHA represent central regulatory hubs that are frequently upregulated, post-translationally modified, or functionally repurposed in cancer cells (Figure 4a).

HK2 catalyzes the first committed step of glucose metabolism and is often tethered to the outer mitochondrial membrane, thereby coupling glycolytic flux to mitochondrial integrity and anti-apoptotic signaling [121]. PFKFB3, through the production of fructose-2,6-bisphosphate, serves as a potent allosteric activator of PFK1, sustaining high glycolytic throughput even under fluctuating nutrient conditions [122]. PKM2, characterized by its dynamic interconversion between high-activity tetramers and low-activity dimers, functions as a metabolic rheostat that balances ATP production with the diversion of glycolytic intermediates into biosynthetic pathways [123]. Meanwhile, LDHA facilitates the conversion of pyruvate to lactate, ensuring efficient regeneration of NAD^+^ and maintaining glycolytic continuity under conditions where mitochondrial oxidation is constrained [124]. Perturbation of these nodes disrupts flux distribution, impairs anabolic processes, and induces metabolic stress, thereby reducing tumor cell viability. The heightened reliance on these enzymatic nodes creates intrinsic points of fragility within the metabolic network. Pharmacological or genetic perturbation of these targets can disrupt flux distribution, impair anabolic capacity, and induce metabolic stress, ultimately compromising tumor cell viability. Thus, enzymatic dependencies constitute a primary layer of actionable vulnerability within rewired glycolysis. These enzymes represent key control points that govern glycolytic flux and integrate metabolic activity with mitochondrial function and survival signaling.

### 6.2. Context-Specific Vulnerabilities

A defining characteristic of glycolytic targeting is its profound context dependency. Distinct oncogenic programs, tissue origins, and microenvironmental constraints collectively shape tumor-specific metabolic states, thereby generating selective and conditionally exploitable vulnerabilities (Figure 4b). These dependencies arise in specific contexts, including hypoxic microenvironments, oncogene-driven metabolic programs such as MYC activation, and conditions of oxidative stress or compromised antioxidant capacity.

Tumors developing in hypoxic microenvironments exhibit a pronounced dependence on glycolysis due to limited OXPHOS capacity, rendering them particularly susceptible to disruptions in glycolytic flux [125]. In MYC-driven cancers, widespread transcriptional amplification of glycolytic and anabolic genes establishes a heightened reliance on glucose metabolism, effectively creating a metabolic bottleneck [126,127]. Furthermore, tumors experiencing elevated oxidative stress or harboring deficiencies in antioxidant systems display increased sensitivity to perturbations in redox homeostasis. Within this framework, redox-sensitive tumors represent a particularly compelling therapeutic opportunity. Glycolysis plays a central role in maintaining intracellular redox balance through regulation of the NAD^+^/NADH ratio and by supporting NADPH generation via the PPP. Disruption of glycolytic flux can therefore destabilize redox homeostasis, leading to the accumulation of ROS and impairment of antioxidant defenses. Consequently, context-specific targeting of glycolysis, particularly in redox-vulnerable settings, provides a rational and mechanistically grounded therapeutic strategy. Thus, disruption of glycolysis in redox-vulnerable tumors destabilizes redox homeostasis, leading to ROS accumulation and impaired antioxidant defenses.

### 6.3. Combination Therapeutic Strategies

Given the remarkable adaptability of tumor metabolism, mono-therapeutic inhibition of glycolysis often results in transient responses and rapid emergence of resistance. Combination strategies have therefore emerged as a more effective paradigm for exploiting metabolic vulnerabilities and achieving durable therapeutic outcomes (Figure 4c). These include combinations with immunotherapy, chemotherapy, and ferroptosis-inducing strategies.

Targeting glycolysis can enhance the efficacy of immunotherapy by reshaping the metabolic landscape of the tumor microenvironment. Inhibition of glycolytic flux alleviates competition for glucose between tumor cells and immune cells while reducing lactate accumulation and extracellular acidification, thereby restoring the metabolic fitness and effector function of tumor-infiltrating lymphocytes [128,129]. In parallel, combining glycolytic inhibition with chemotherapy can potentiate cytotoxic effects by amplifying metabolic stress, depleting biosynthetic intermediates, and impairing the capacity of cancer cells to repair damage [130,131].

Particularly promising is the integration of glycolytic targeting with ferroptosis induction. By limiting NADPH production and disrupting redox homeostasis, inhibition of glycolysis compromises the cellular antioxidant network and promotes lipid peroxidation. This effectively lowers the threshold for ferroptotic cell death and enhances the efficacy of ferroptosis-inducing agents [132,133]. This effect is driven by reduced NADPH availability, disruption of redox balance, and enhanced lipid peroxidation. Such combinatorial approaches leverage the intrinsic coupling between glycolysis, redox regulation, and ferroptosis sensitivity, offering a conceptually coherent and mechanistically grounded strategy for cancer therapy.

### 6.4. Resistance and Metabolic Compensation

Despite these therapeutic opportunities, targeting glycolysis is fundamentally challenged by the extensive plasticity of cancer metabolism and the activation of compensatory pathways. In response to glycolytic inhibition, tumor cells can rewire their metabolic networks to engage alternative sources of energy and biosynthetic precursors, thereby maintaining cellular homeostasis under therapeutic pressure (Figure 4d). These adaptations commonly include increased reliance on mitochondrial OXPHOS, enhanced glutaminolysis to replenish tricarboxylic acid cycle intermediates, and activation of fatty acid oxidation pathways [7,134]. Notably, emerging evidence suggests that distinct glycolytic perturbations may preferentially engage specific compensatory routes. For example, inhibition of LDHA disrupts NAD^+^ regeneration and redox balance, thereby promoting a compensatory shift toward glutaminolysis to sustain TCA cycle flux and redox homeostasis [135]. In contrast, blockade of lactate export via MCT1 inhibition leads to intracellular lactate accumulation and metabolic stress, which can increase reliance on mitochondrial oxidative metabolism [136]. Inhibition of upstream glycolytic nodes, such as HK2, reduces glycolytic flux and can shift metabolic dependence toward mitochondrial respiration or alternative substrates, including fatty acid oxidation [137].

Beyond these dominant compensatory routes, metabolic adaptation is not strictly linear. Inhibition of a single glycolytic node may simultaneously engage multiple alternative pathways, depending on cellular context and metabolic state [15]. This reflects the highly interconnected nature of metabolic networks, in which flux redistribution rather than pathway switching per se underlies adaptive resistance. Such compensatory mechanisms are often reinforced by signaling and transcriptional feedback loops that re-establish metabolic equilibrium. These considerations underscore the limitations of single-pathway targeting and highlight the need for therapeutic strategies that account for metabolic diversity and adaptability. Rational combination therapies that simultaneously disrupt multiple metabolic nodes, or that target upstream regulators governing metabolic flexibility, may be required to overcome resistance. Accordingly, effective therapeutic strategies require disruption of multiple metabolic nodes or targeting of upstream regulators that govern metabolic flexibility and plasticity.

Taken together, glycolysis-targeted therapy is shaped by enzymatic dependencies, context-specific vulnerabilities, combinatorial strategies, and adaptive metabolic compensation, collectively determining therapeutic efficacy and resistance (Figure 4).

### 6.5. Translational and Clinical Targeting of Glycolysis

Despite extensive mechanistic insights into glycolytic reprogramming, its translation into effective clinical strategies remains limited and heterogeneous. While early-stage tumorigenesis is characterized by well-defined metabolic rewiring driven by oncogenic signaling, the therapeutic exploitation of glycolysis is constrained by metabolic plasticity, compensatory pathway activation, and context-dependent dependencies.

Current strategies targeting glycolysis can be broadly categorized into four major approaches: (i) inhibition of glucose utilization, (ii) targeting rate-limiting glycolytic enzymes, (iii) disruption of lactate production and export, and (iv) metabolic reprogramming toward OXPHOS. To provide a structured and translationally grounded overview, we have systematically summarized representative glycolysis-targeting agents, including their molecular targets, mechanisms of action, development stage, and associated biomarkers, in Table 1.

Among these, glucose analogs such as 2-deoxy-D-glucose competitively inhibit hexokinase activity and glycolytic flux, although their clinical efficacy has been modest due to compensatory metabolic adaptations [138,139]. More selective approaches include inhibitors targeting key enzymes such as PFKFB3, PKM2, and LDHA, which regulate glycolytic flux, biosynthetic branching, and redox balance [140,141]. Notably, several glycolysis-targeting agents have progressed into early-phase clinical evaluation. For instance, monocarboxylate transporters (MCTs), such as AZD3965, have progressed into clinical trials, particularly in tumors with high MCT1 expression and low MCT4 levels, highlighting the importance of biomarker-guided patient selection [142,143]. In addition, metabolic modulators such as dichloroacetate, which promotes pyruvate entry into the mitochondria by inhibiting pyruvate dehydrogenase kinase, represent an alternative strategy to counteract the Warburg phenotype by restoring oxidative metabolism [144]. Importantly, the clinical and preclinical landscape summarized in Table 1 highlights that therapeutic responses to glycolytic inhibition are highly context-dependent and influenced by tumor genotype (e.g., MYC, KRAS, HIF-1α activation), metabolic flexibility, and microenvironmental constraints [145]. These observations emphasize the need for integrated biomarker-driven strategies, including metabolic imaging, transcriptomic profiling, and single-cell analyses, to identify patient subgroups most likely to benefit from glycolysis-targeted therapies.

Collectively, these approaches highlight that therapeutic targeting of glycolysis aims to exploit metabolic dependencies, disrupt tumor-supportive metabolic interactions, and restore metabolic balance in cancer cells.

**Table 1:** Glycolysis-targeting agents in preclinical and clinical development.

Category	Agent	Target	Mechanism	Stage	Cancer Types	Biomarkers of Sensitivity	Ref. (PMID/NCT)
Glucose analog	2-DG	HK2/glycolysis	Competitive inhibition of glucose metabolism	Phase I/II	cerebral gliomas, solid tumors	High glucose uptake, HK2 expression	NCT00096707 PMID: 8641905 [139]
PFKFB3 inhibitor	PFK-158	PFKFB3	Reduces fructose-2,6-bisphosphate, suppressing glycolytic flux	Phase I	Solid tumors	High PFKFB3 expression	NCT02044861
PKM2 modulator	TEPP-46	PKM2	Stabilizes active tetrameric PKM2	Preclinical	Multiple cancers	PKM2-dependent tumors	PMID: 29207616 [146], PMID: 31527446 [140]
LDHA inhibitor	FX-11	LDHA	Blocks pyruvate-to-lactate conversion	Preclinical	Lymphoma, pancreatic cancer	LDHA-high tumors	PMID: 27919448 [147], PMID: 33966212 [141]
LDHA inhibitor	GNE-140	LDHA	Potent LDHA inhibition	Preclinical	pancreatic cancer	LDHA-high tumors	PMID: 27479743 [148]
MCT1 inhibitor	AZD3965	MCT1	Blocks lactate transport	Phase I	Lymphoma	MCT1-high/MCT4-low	NCT01791595
Metabolic modulator	DCA	PDK	Activates PDH, shifts to OXPHOS	Phase I/II	Glioma	High PDK activity	NCT01111097 NCT00540176 NCT05120284
Enolase inhibitor	HEX	ENO1	Synthetic lethality in ENO1-deleted tumors	Preclinical	Glioma	ENO1 deletion	PMID: 33230295 [149]

## 7. Emerging Technologies and Conceptual Advances

A deeper and more mechanistic understanding of glycolytic rewiring in cancer is increasingly driven by the convergence of advanced experimental and computational technologies that enable high-resolution, dynamic, and systems-level interrogation of tumor metabolism. Traditional approaches, which largely rely on bulk measurements of metabolite abundance or gene expression, are insufficient to capture the complexity, heterogeneity, and temporal dynamics of metabolic reprogramming. In contrast, emerging technologies are redefining glycolysis not as a static pathway but as a highly context-dependent and spatially organized network. These advances are fundamentally reshaping how metabolic heterogeneity, plasticity, and therapeutic vulnerabilities are conceptualized and exploited, thereby opening new avenues for precision targeting cancer metabolism.

Single-cell metabolomics represents a breakthrough in resolving metabolic diversity at unprecedented resolution. By profiling metabolites or metabolic proxies at the level of individual cells, this approach uncovers previously masked heterogeneity in glycolytic activity within tumors. Importantly, it enables the identification of distinct metabolic subpopulations, including rare but clinically significant cell types such as CSCs or therapy-resistant cells [150,151,152]. These populations often exhibit unique metabolic configurations, such as differential reliance on glycolysis versus OXPHOS, which cannot be captured by bulk analyses. As a result, single-cell approaches provide critical insights into how glycolytic rewiring contributes to intratumoral heterogeneity, lineage plasticity, and therapeutic resistance. Complementing this cellular resolution, spatially resolved omics technologies introduce a critical architectural dimension to the study of tumor metabolism. Spatial transcriptomics and metabolomics allow the mapping of glycolytic activity, lactate gradients, and nutrient distribution within intact tumor tissues, thereby linking metabolic states to their microenvironmental context [153,154]. These approaches reveal that glycolysis is often spatially compartmentalized, with highly glycolytic, hypoxia-adapted cells localized to poorly vascularized tumor regions, while more oxidative phenotypes predominate in oxygen-rich niches. Such spatial heterogeneity not only reflects microenvironmental constraints but also supports functional interactions between tumor cell populations, including metabolic symbiosis and metabolite exchange.

In parallel, metabolic flux analysis has emerged as an indispensable tool for capturing the dynamic behavior of glycolytic networks. Unlike static measurements of metabolite levels, isotope tracing approaches using labeled substrates enable direct quantification of metabolic pathway activity and carbon flow [155,156]. This allows for precise dissection of how glycolytic intermediates are partitioned between energy production, biosynthetic pathways, and redox maintenance. Flux-based analyses are particularly valuable for revealing context-dependent rerouting of metabolism during tumor progression or in response to therapeutic perturbations, thereby providing a functional readout of metabolic plasticity that cannot be inferred from steady-state data alone.

Advances in computational modeling and artificial intelligence (AI) are further accelerating the translation of these complex datasets into actionable biological insights [157,158]. By integrating multi-omics layers, including genomics, transcriptomics, proteomics, and metabolomics, AI-driven frameworks can infer context-specific metabolic dependencies and predict vulnerabilities within glycolytic networks. Constraint-based modeling and machine learning approaches also enable simulation of metabolic responses to genetic or pharmacological perturbations, facilitating the rational design of combination therapies [159,160]. Importantly, these computational strategies provide a unifying platform to bridge experimental observations with predictive, system-level understanding of tumor metabolism.

These emerging concepts and technologies are driving a transition toward personalized metabolic therapy. By integrating single-cell resolution, spatial context, and dynamic flux information, it becomes increasingly feasible to define patient-specific metabolic states and identify context-dependent vulnerabilities within glycolytic pathways. This transformation not only deepens our mechanistic understanding of glycolytic rewiring but also lays the foundation for the development of more precise and effective therapeutic strategies targeting cancer metabolism.

## 8. Conclusions and Future Perspectives

Glycolysis in cancer should no longer be viewed as a static metabolic hallmark but rather as a dynamically rewired process that evolves throughout tumorigenesis. From early transformation to advanced progression, glycolytic pathways are continuously reshaped by oncogenic signaling, epigenetic regulation, and microenvironmental pressures, enabling cancer cells to coordinate energy production with biosynthesis, redox balance, and adaptive signaling. This dynamic rewiring underscores the role of glycolysis as a central organizing framework in tumor biology rather than a mere metabolic pathway.

While such rewiring provides substantial advantages for tumor growth, survival, and plasticity, it also imposes intrinsic constraints that give rise to exploitable vulnerabilities. The dependence on specific enzymatic nodes, the need to maintain redox homeostasis, and the integration with cell death and immune regulatory pathways collectively create context-dependent liabilities. These vulnerabilities are not fixed but emerge from the evolving metabolic state of cancer cells, highlighting the importance of understanding glycolysis within its temporal and environmental context.

Looking forward, therapeutic strategies should move beyond uniform inhibition of glycolysis and instead focus on exploiting these dynamically acquired vulnerabilities. Integrating multi-dimensional insights, including metabolic flux, spatial organization, and cellular heterogeneity, will be essential for identifying actionable targets and designing effective interventions. In this context, coupling glycolytic targeting with strategies that modulate redox balance, immune responses, or ferroptosis may offer particularly promising avenues. Ultimately, a deeper, systems-level understanding of glycolytic rewiring will pave the way for precise, context-aware, and durable metabolic therapies in cancer.

## Data Availability

No data was generated in this study.

## Abbreviations

The following abbreviations are used in this manuscript:

KRAS	Kirsten rat sarcoma
MYC	Myelocytomatosis
HK2	Hexokinase 2
PFK	Phosphofructokinase
LDHA	Lactate dehydrogenase A
PI3K	Phosphoinositide 3-kinase
AKT	Ak strain transforming
G6P	Glucose-6-phosphate
ROS	Reactive oxygen species
PPP	Pentose phosphate pathway
HIF-1α	Hypoxia-inducible factor 1α
PTMs	Post-translational modifications
PKM2	Pyruvate kinase M2
OXPHOS	Oxidative phosphorylation
CSCs	Cancer stem cells
PFKFB3	phosphofructokinase-2/fructose-2,6-bisphosphatase 3
AI	Artificial intelligence
